# Mechanical Properties and Fracture Behavior of Crumb Rubber Basalt Fiber Concrete Based on Acoustic Emission Technology

**DOI:** 10.3390/s20123513

**Published:** 2020-06-21

**Authors:** Hanbing Liu, Wenjun Li, Guobao Luo, Shiqi Liu, Xiang Lyu

**Affiliations:** College of Transportation, Jilin University, Changchun 130025, China; lhb@jlu.edu.cn (H.L.); wenjun18@mails.jlu.edu.cn (W.L.); sqliu17@mails.jlu.edu.cn (S.L.); lvxiang18@mails.jlu.edu.cn (X.L.)

**Keywords:** crumb rubber, basal fiber, orthogonal test, mechanical properties, acoustic emission, fracture behavior

## Abstract

Basalt fiber and crumb rubber, as excellent road material modifiers, have great advantages in improving the mechanical properties and fracture behavior of concrete. Acoustic emission (AE) is a nondestructive testing and real-time monitoring technique used to characterize the fracture behavior of concrete specimens. The object of this paper is to investigate the effects of crumb rubber replacement rate, basalt fiber content and water–binder ratio on the mechanical properties and fracture behavior of crumb rubber basalt fiber concrete (CRBFC) based on orthogonal test. The fracture behavior of a CRBFC specimen under three-point flexural conditions was monitored by AE technology and the relative cumulative hit (*RCH*) was defined to characterize the internal damage degree of CRBFC. The experimental results showed that, considering the mechanical strength and fracture damage behavior of CRBFC, the optimal crumb rubber replacement rate, basalt fiber content and water–binder ratio are 10%, 2 kg/m^3^ and 0.46, respectively. In addition, it was found that AE parameters can effectively characterize the fracture behavior of CRBFC. The fracture stages of CRBFC can be divided according to the cumulative AE hits and counts. AE amplitude value can be used as an early warning of CRBFC specimen fracture. Moreover, the fracture mode can be identified by RA and average frequency (AF) values variation during the loading process.

## 1. Introduction

In recent years, the output of waste tires has increased greatly with the rapid development of the automobile industry. Massive quantities of waste rubber are generated every year, leading to increasingly serious environmental pollution [1,2]. The waste rubber is not biodegradable and excessive waste rubber causes secondary pollution, thus increasing the burden on the ecological environment [3]. At present, less than 50% of waste rubber is recycled and the rest is buried, which causes the waste of land resources [4,5]. How to recycle and reuse the waste rubber has aroused the widespread concern of the government and the public. The recycling technology for waste rubber has gradually matured with the development of industrial technology and waste rubber has been used in the fields of manufacturing tires, modified asphalt and elastic floor tiles. In addition, adding crumb rubber, produced by waste rubber tires, into concrete can realize the recycling and reusing of waste rubber, meeting the requirements of environmentally sustainable development [6,7]. The addition of crumb rubber can improve the freeze–thaw durability of concrete [8] and the resistance to chloride ion penetration [9]. In addition, the replacement of fine aggregate with crumb rubber can reduce the weight of concrete and improve the toughness and cracking resistance of concrete [10,11]. However, the hydrophobicity of crumb rubber poses a great challenge to the bonding strength between crumb rubber and cement. Studies on the mechanical properties of rubber-modified concrete show that the compressive strength, flexural strength, splitting tensile strength and elastic modulus of concrete decrease with the increase in crumb rubber replacement rate [12,13]. Hence, it is necessary to improve the mechanical properties of crumb rubber concrete. 

Adding fibers into concrete can effectively improve the mechanical properties and durability of concrete. Three-dimensional randomly distributed fibers can inhibit the initiation and expansion of cracks, reduce the stress concentration near cracks and improve the toughness of concrete [14,15]. The common fiber in the field of fiber-reinforced concrete is steel fiber. Steel fiber and crumb rubber have a positive synergistic effect, which enhances the strength and strain capacity of concrete [16,17]. However, steel fiber with high content converges into a ball and the steel fiber is sensitive to corrosion, thus reducing the performance of concrete. Basalt fiber, as an alternative fiber, is an economical and environmentally friendly inorganic fiber obtained from natural basalt by melting and stretching at high temperatures [18]. The basalt fiber not only has high tensile strength but also has great acid and alkaline resistance, high temperature resistance and corrosion resistance [19]. In addition, the chemical properties of basalt fiber and cement are similar and the bonding strength between basalt fiber and cement is great [20]. Basalt fiber is promising in the application of fiber-reinforced materials due to its remarkable performance and environmentally friendly fabrication process. Branston et al. [21] investigated the effects of two kinds of basalt fiber (bundle dispersion fibers and minibars) on the mechanical properties of concrete; the result showed that two kinds of basalt fiber can significantly improve the flexural strength of concrete and only minibars can enhance the properties after cracking. Algin et al. [22] researched the effect of basalt fiber content on the mechanical properties of concrete and obtained the optimal content of basalt fiber. Niu et al. [23] reported that basalt fiber can effectively optimize the pore structure of concrete and inhibit the penetration of chloride ions. 

Acoustic emission (AE) is a nondestructive testing and real-time monitoring technology, which has been used to analyze the actual failure state and fracture behavior of concrete [24]. The elastic wave generated by the internal damage of concrete and collected by AE sensors is used to investigate the actual failure state of the concrete [25]. The recorded AE signal parameters change with the initiation and expansion of cracks during the fracture process of concrete, and the fracture behavior can be detected from the beginning of cracks to the final failure [26,27,28]. Moreover, AE technology has the ability to accurately locate the damage source, characterize the strength in the fracture process and identify the damage mode [29,30]. Previous studies have shown that AE parameters can effectively reflect the stage characteristics in the concrete fracture process [31]. Shiotani et al. [32] proposed an improved b-value (Ib-value) method based on the statistical information of events. The Ib-value has been used in the field of concrete materials to evaluate the fracture state of concrete and the generation of major cracks [33]. Chen et al. [34] adopted AE technology to obtain the main internal and external damage positions in the fracture change stage, determine the position of the three-dimensional AE source, and evaluate the characteristics of the fracture process zone of concrete through the change in the source position. Abouhussien et al. [35] evaluated the structural damage of concrete by AE signal strength analysis and detected the initiation and expansion of microcracks and macroscopic cracks through AE analysis. Xargay et al. [36] analyzed the fracture mode of concrete by RA and average frequency (AF) values.

In this paper, the effects of water–binder ratio (0.43, 0.46 and 0.49), basalt fiber content (2 kg/m^3^, 4 kg/m^3^ and 6 kg/m^3^) and crumb rubber replacement rate (5%, 10% and 15%) on the mechanical properties and fracture behavior of crumb rubber basalt fiber concrete (CRBFC) have been investigated by orthogonal test. The purpose of this study is to reduce the production cost of concrete, improve the mechanical properties of concrete and realize the recycling and reuse of waste rubber. In addition, a three-point flexural test of CRBFC was carried out with AE technology. The fracture behavior of CRBFC was evaluated based on visual analysis of AE parameters, and AE characteristic parameters were used as an early warning of concrete fracture. Moreover, the fracture mode of CRBFC was investigated by the AE representative analysis method (RA and AF values), and the application of AE methods to concrete structures was certified.

## 2. Materials and Methods

### 2.1. Materials

**Figure 1:**
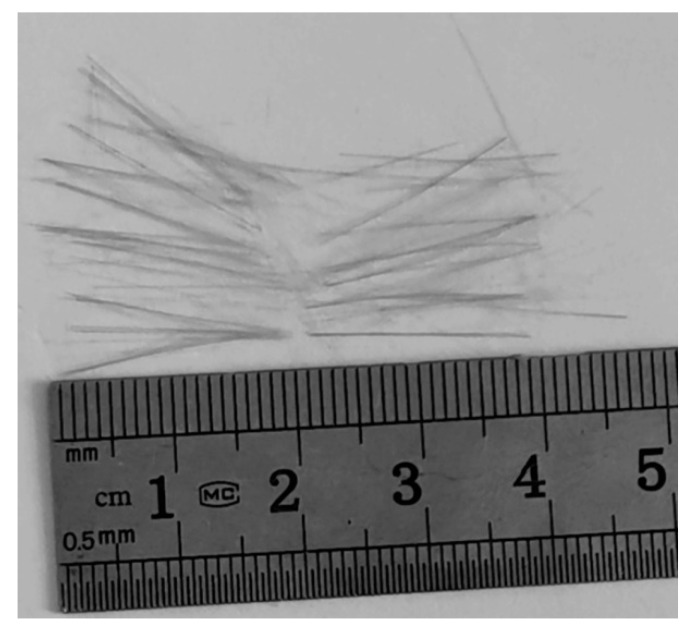
Basalt fiber.

Type P.II Portland cement with the strength of 52.5 MPa, produced by Yatai Cement Co., Ltd., Jilin, was selected as the cementitious material in this study. The physical properties and chemical composition of cement are shown in Table 1 and Table 2. A natural aggregate with continuous gradation from 5 mm to 25 mm was used as the coarse aggregate. River sand with particles of 0.1–4.75 mm was used as the fine aggregate and the fineness modulus was 2.7. Crumb rubber with particles of 0.1–4.75 mm was used and the fineness modulus was 2.35. NaOH (chemical purity, 95.38%) was used to modify crumb rubber. Chopped basalt fiber obtained from Anjie Composite Material Co., Ltd., Haining, was used and its physical properties are given in Table 3. The appearance of basalt fiber is shown in Figure 1. The mixed water was tap water.

### 2.2. Pre-Modification of Crumb Rubber

The hydrophobic nature of crumb rubber leads to the generation of pore structures between crumb rubber and cement, thus increasing the porosity of the concrete. In addition, the bonding strength between crumb rubber and cement paste is weak because of the hydrophobic nature of crumb rubber, which will further reduce the strength of the concrete [37,38].

The aging crumb rubber contains carboxyl groups (–COOH) and the hydrogen ions in carboxyl groups (–COOH) can be replaced by sodium ions and form carboxylates (–COONa). Carboxylates (–COONa) can provide a weak alkali environment around crumb rubber and improve cement hydration near crumb rubber [39]. In addition, the high hydrophilic carboxylates (–COONa) can increase the hydrophilic properties of crumb rubber and reduce the interfacial porosity between crumb rubber and cement paste. Hence, crumb rubber modified by sodium hydroxide solution can improve the strength of concrete [40].

The procedures for sodium hydroxide solution pre-modified crumb rubber were as follows: (1) crumb rubber was soaked in 0.25 M sodium hydroxide solution for 24 h at room temperature; (2) crumb rubber treated with sodium hydroxide solution was washed with tap water until the pH of the washing water was close to neutral; (3) crumb rubber was air dried at room temperature.

### 2.3. Orthogonal Experimental Design

Orthogonal experimental design is a method to study multi-factor and multi-level experiments. According to the orthogonality, a few experimental schemes with enhanced representativeness were selected evenly from the whole experimental group and high-quality output can be obtained from the few experimental results [41]. The selection of an orthogonal table is the key to orthogonal experimental design. The orthogonal table is denoted as L_n_(r^m^), where *L* refers to the orthogonal table, *n* refers to the number of experiments, *m* refers to the maximum number of factors, and *r* refers to the number of levels used by each factor.

The water–binder ratio, crumb rubber replacement rate and basalt fiber content have great influence on the mechanical properties of CRBFC. These three parameters were selected as orthogonal test factors and each factor had three levels, as shown in Table 4. The orthogonal table with four factors and three levels was selected for orthogonal experimental design. The specific experimental design is shown in Table 5.

Range analysis is a statistical method to judge the sensitivity of experimental results to each factor in an orthogonal test. The effect of each factor on the experimental result was judged by comparing the magnitude of the range. The larger the range, the greater the influence of each factor on the experimental results. The specific calculation process of range analysis is shown in Equations (1) and (2):

(1) $$k_{xi}=K_{xi}/3$$

(2) $$R_{x}=\max\{k_{x1},k_{x2},k_{x3}\}-\min\{k_{x1},k_{x2},k_{x3}\}$$

where *K_xi_* refers to the sum of experimental results corresponding to the level *i* of factor *x*, *k_xi_* refers to the average of experimental results corresponding to the level *i* of factor *x*, and *R_x_* refers to the range value of experimental results corresponding to factor *x*.

Variance analysis based on the *F*-test was used to verify the creditability of range analysis and accurately estimate the importance of each factor for the experimental results. The null hypothesis is that the given factor has no impact on the experimental results. The null hypothesis is rejected if the obtained *F* value is equal to or higher than the critical *F* value at the significant level. The higher the *F* value, the more significant the influence of the given factor on the experimental results is [42]. The calculation process of the variance analysis is shown in Equations (3)–(5):

(3) $$MS_{f}=\frac{SS_{f}}{d_{f}}$$

(4) $$MS_{e}=\frac{SS_{e}}{d_{e}}$$

(5) $$F=\frac{MS_{f}}{MS_{e}}$$

where *SS_f_* refers to the sum of square deviations of each factor. *SS_e_* refers to the sum of square deviations of error. *d_f_* refers to the degrees of freedom of each factor. *d_e_* refers to the degrees of freedom of error. *MS_f_* refers to the mean square of each factor. *MS_e_* refers to the mean square of error. *F* refers to the *F* value.

### 2.4. Preparation of CRBFC

The crumb rubber was added into the concrete to replace fine aggregate with an equivalent volume method. Unmodified crumb rubber was mixed into concrete as the control group to evaluate the effect of crumb rubber modified by sodium hydroxide solution on the strength of CRBFC. In addition, a control group without added crumb rubber and basalt fiber was prepared to evaluate the beneficial effects of crumb rubber and basalt fiber on the strength of concrete. The mix proportion of CRBFC is shown in Table 6.

The dispersion of crumb rubber and basalt fiber has a great effect on the strength of concrete. During the mixing process, crumb rubber and basalt fiber were added into the mixture step by step, and the specific mixing steps were as follows. (1) Put the coarse aggregate and fine aggregate into the blender and stir for 1 min. (2) Put the basalt fiber into the blender and stir for 2 min. (3) Put the premixed cement and crumb rubber mixture into the blender and stir for 1 min. (4) Put the water into the blender and stir for 3 min. The 100 × 100 × 100 mm and 100 × 100 × 400 mm specimens were casted. All the specimens were de-moulded after 24 h and cured for 28 d in a standard-cured room with a relative humidity of 95% and a temperature of 20 ± 2 °C.

### 2.5. Test Methods for Mechanical Properties

**Figure 2:**
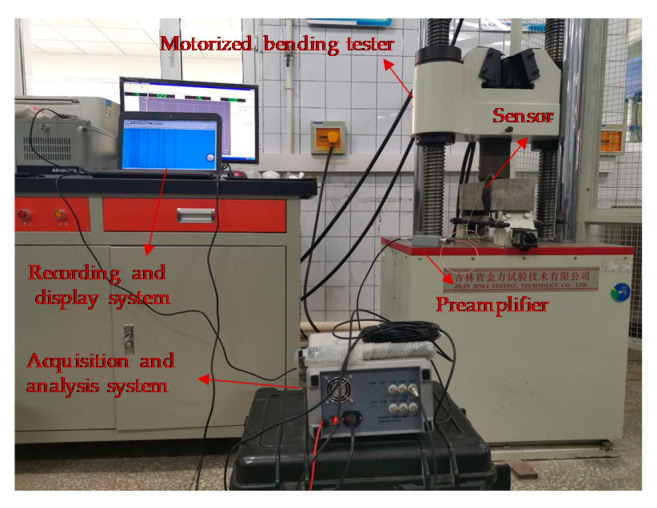
The specific experimental device.

The compressive strength and flexural strength of CRBFC were tested according to national standard GB/T50081–2002 [43]. The 100 × 100 × 100 mm specimen was used for the compressive strength test. The 100 × 100 × 400 mm specimen was used for the flexural strength test. The flexural strength was tested by a three-point bending test, and the distance between the two fulcrum points was 300 mm. The AE technology was used to real-time monitor the fracture behavior of CRBFC under three-point flexural conditions. The specific experimental device is shown in Figure 2.

### 2.6. AE Method

**Figure 3:**
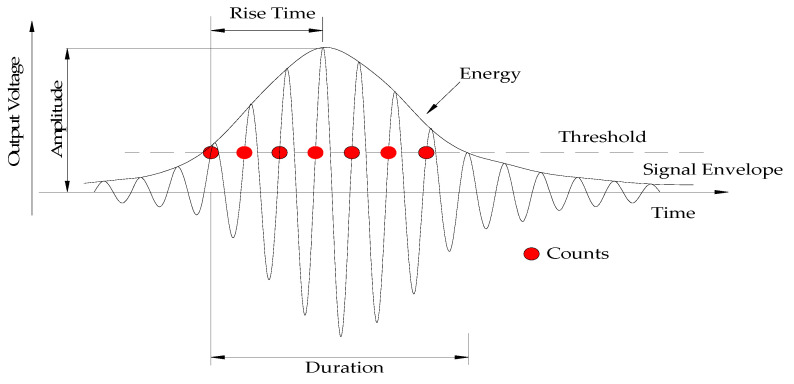
Basic parameters of AE.

The variation in AE signal parameters is related to change in the internal structure of concrete. The elastic wave generated by damage to the internal structure of concrete propagates to the surface of the AE sensor and converts it into an electrical signal. The electrical signal is processed and calculated by the acquisition and analysis system into a set of signal parameters to characterize the digitized AE waveform. AE signal parameters collected by the AE measurement system are shown in Figure 3. The threshold value set by the user is the key AE parameter and has an effect on the accuracy of the data. AE amplitude refers to the maximum amplitude value of the corresponding event signal waveform, and the unit is decibels (dB). Rise time refers to the time interval from when the event signal first crosses the threshold until it reaches the maximum amplitude, and the unit is microseconds (μs). Duration refers to the time interval between when the event signal first crosses the threshold and the final drops to the threshold, and the unit is microseconds (μs). AE energy refers to the elastic energy released by AE events, and the unit is mv∙μs. Counts refers to the number of AE amplitudes that cross the threshold.

In order to classify cracking modes, AE parameters are applied to calculate RA and AF values. AF is defined as count divided by duration; RA is defined as rise time divided by amplitude [44]. The specific calculation equations are as follows:

(6) $$RA=\frac{Rise\;Time}{Amplitude},ms/V$$

(7) $$AF=\frac{Counts}{Duration},kHz$$

The fracture modes can be divided into shear mode and tensile mode. Compared with the tensile crack, the shear crack has a higher RA value and lower AF value, which is related to the energy wave released by the crack. The tensile crack releases energy in the form of a longitudinal wave, which has a faster propagation speed than a shear wave, with a large amplitude and short rise time. The shear crack releases energy mainly in the form of a shear wave, which has a slower propagation speed leading to a longer duration and rise time. 

**Figure 4:**
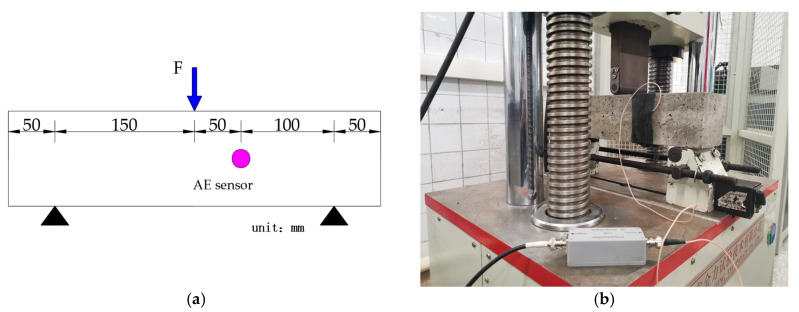
The arrangement of AE sensors: (**a**) schematic diagram; (**b**) specific arrangement.

In the three-point flexural test, the fracture behavior of CRBFC specimens was detected by the SAEU2S–6 AE acquisition and analysis system produced by Shenghuaxingye Technology Co., Ltd. (Beijing, China). A SR150M 150kHz resonant AE sensor produced by Shenghuaxingye Technology Co., Ltd. (Beijing, China) with a frequency range of 60–400 kHz was fixed on the side of the specimen through an elastic fixing band. Vaseline was used as a coupling agent to eliminate the air in the contact interface. The lead breaking test was used to ensure the AE sensor was contacted well. The specific arrangement of the AE sensor is shown in Figure 4. The preamplifier gain was set to 40 dB. The threshold value of automatic exposure detection was set to 40 dB to filter out environmental noise and the low-pass and high-pass filters were set to 20 kHz and 400 kHz, respectively, to filter out electrical noise. The sampling frequency was 5MSPS. In order to ensure the integrity and accuracy of the signal acquisition, the peak definition time, hit definition time and hit lock time were set to 50 μs, 100 μs and 300 μs, respectively.

## 3. Results and Analysis

### 3.1. Orthogonal Test

The experimental results of the compressive strength and flexural strength tests are shown in Table 7. The control group B1 was compared with the third group of orthogonal tests to evaluate the effect of 0.25 M sodium hydroxide solution modification on the strength of concrete. It can be seen from Table 7 that the compressive strength and flexural strength of concrete in the control group are 5.6% and 17.5% lower than those in the third group of orthogonal tests, respectively. This indicates that the 0.25 M sodium hydroxide solution modification can significantly improve the strength of CRBFC. Table 7 shows that the compressive strength and flexural strength of the concrete in the control group C are lower than those in the test group; it indicates that crumb rubber and basalt fiber can significantly improve the strength of concrete.

#### 3.1.1. Compressive Strength

**Figure 5:**
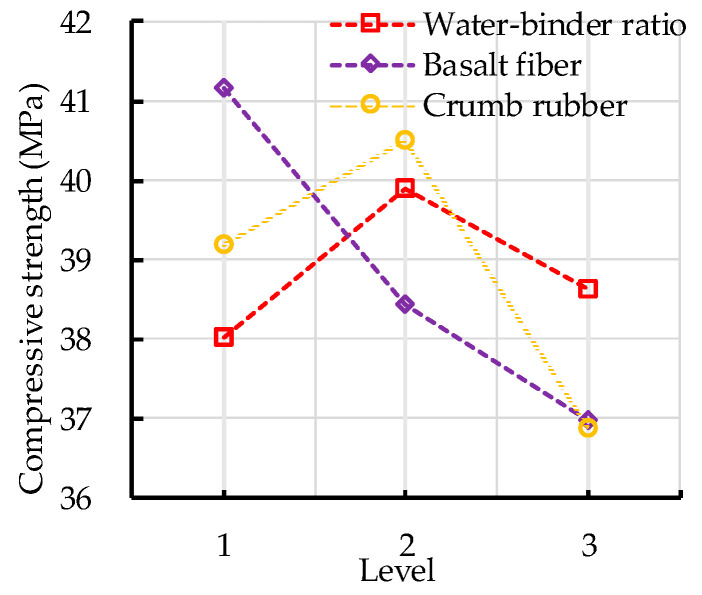
Effects of water–binder ratio, basalt fiber content and crumb rubber replacement rate on the compressive strength of CRBFC.

The range analysis of the compressive strength of CRBFC is shown in Table 8. The range values of water–binder ratio, basalt fiber content, crumb rubber replacement rate and error were calculated, and the error range is the minimum. This indicates that the effect of error on the experiment results is less than that of the three independent factors. It can be seen from Table 8 that the basalt fiber content has the greatest effect on the compressive strength of CRBFC, followed by crumb rubber replacement rate and water–binder ratio. The effects of water–binder ratio, basalt fiber content and crumb rubber replacement rate on the compressive strength of CRBFC are shown in Figure 5. The compressive strength of CRBFC increases first and then decreases with the increase in water–binder ratio. The compressive strength of CRBFC decreases with the increase in basalt fiber content. The appropriate amount of fiber submits a three-dimensional random distribution, which limits the transverse deformation of concrete under compressive conditions. However, excessive fibers will absorb the water in the concrete mixture, which restricts the hydration of cement and reduces the compressive strength of concrete. The compressive strength of CRBFC increases first and then decreases with the increase in crumb rubber replacement rate. When the crumb rubber replacement rate is lower than 10%, the positive effect of sodium hydroxide solution modification is stronger than the negative effect of crumb rubber incorporation; when the crumb rubber replacement rate is higher than 10%, the negative effect of crumb rubber incorporation is stronger and reduces the compressive strength of CRBFC. In addition, it can be seen from Table 8 and Figure 5 that the optimal levels of water–binder ratio, basalt fiber content and crumb rubber replacement rate are 0.46, 2 kg/m^3^ and 10%, respectively.

The variance analysis of the compressive strength of CRBFC is shown in Table 9. As seen in Table 9, there are three *F* critical values that define the significance of each independent factor: if the *F* value is greater than 19.0, the effect of the factor is the most significant; if the *F* value is between 9.0 and 19.0, the effect of the factor is significant; if the *F* value is between 3.0 and 9.0, the effect of the factor is less significant; if the *F* value is less than 3.0, the effect of the factor is least significant. Table 9 shows that the *F* values of basalt fiber content and crumb rubber replacement rate are between 9.0 and 19.0, which indicates that these two factors have significant effects on the compressive strength of CRBFC. The *F* value of basalt fiber content is greater than that of crumb rubber replacement rate, which means the effect of basalt fiber content is more significant than that of crumb rubber. The *F* value of the water–binder ratio is between 3.0 and 9.0, which indicates that the water–binder ratio has a less significant effect on the compressive strength of CRBFC. The results are consistent with that of range analysis.

#### 3.1.2. Flexural Strength

**Figure 6:**
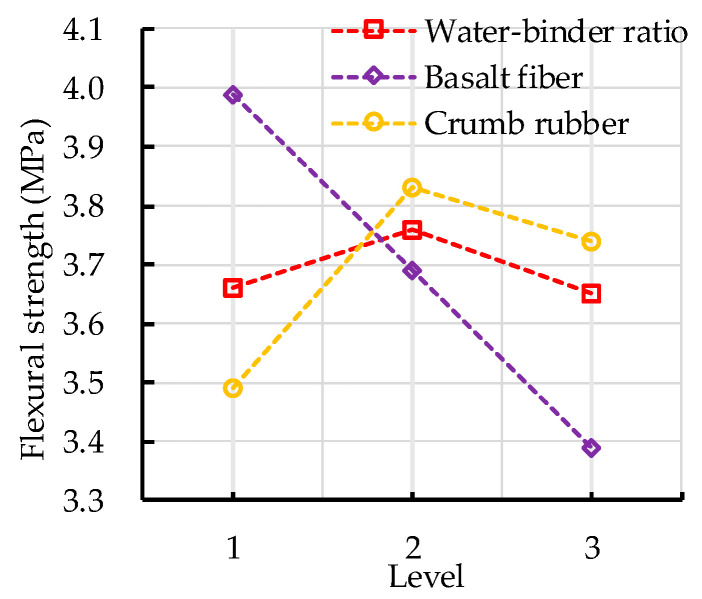
Effects of water–binder ratio, basalt fiber content and crumb rubber replacement rate on flexural strength of CRBFC.

The range analysis of the flexural strength of CRBFC is shown in Table 10. Table 10 shows that the error has the least impact on the flexural strength of CRBFC, and the basalt fiber content has the greatest impact on the flexural strength of CRBFC, followed by the crumb rubber replacement rate and water–binder ratio. The effects of water–binder ratio, basalt fiber content and crumb rubber replacement rate on the flexural strength of CRBFC are shown in Figure 6. The flexural strength of CRBFC increases first and then decreases with the increase in water–binder ratio. The flexural strength of CRBFC decreases with the increase in basalt fiber content. The appropriate amount of basalt fiber can inhibit the initiation and expansion of cracks, reduce the stress concentration near cracks and make the stress distribution more uniform, thus improving the flexural strength of concrete. However, excessive basalt fibers will destroy the compacted structure of concrete, reducing the flexural strength of concrete. The flexural strength of CRBFC increases first and then decreases with the increase in crumb rubber replacement rate. The reason is that with the increase in the crumb rubber replacement rate the negative effects of hydrophobicity and low strength of crumb rubber are gradually stronger than the positive effects of sodium hydroxide solution modification. In addition, it can be seen from Table 10 and Figure 6 that the optimal levels of water–binder ratio, basalt fiber content and crumb rubber replacement rate are 0.46, 2 kg/m^3^ and 10%, respectively.

The variance analysis of the flexural strength of CRBFC is shown in Table 11. The significance level judgment of flexural strength is the same as compressive strength. It can be seen from Table 11 that the *F* value of basalt fiber content is greater than 19.0; this indicates that basalt fiber content has the most significant effect on the flexural strength of CRBFC. The *F* value of the crumb rubber replacement rate is between 9.0 and 19.0, which means the crumb rubber replacement rate has a significant effect on the flexural strength of CRBFC. The *F* value of the water–binder ratio is less than 3.0, which indicates that the effect of the water–binder ratio is least significant. The results are consistent with those of range analysis.

#### 3.1.3. AE Parameters

**Figure 7:**
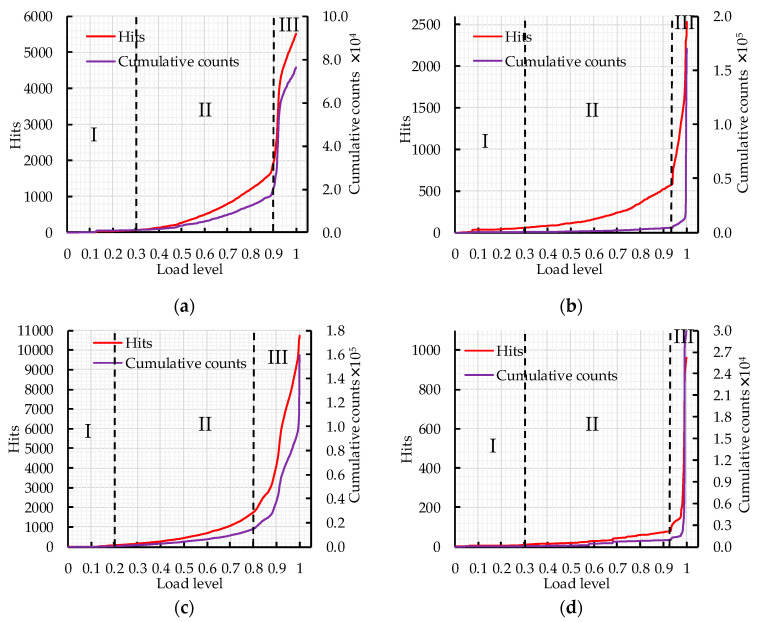

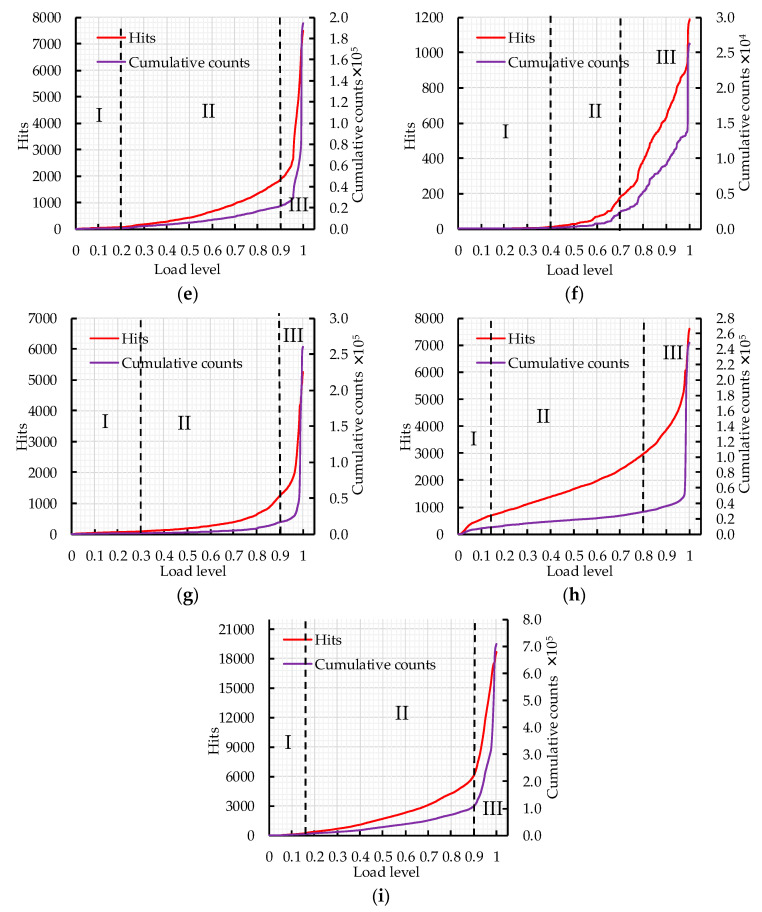
The curve of cumulative AE hits and counts versus load level: (**a**) A1; (**b**) A2; (**c**) A3; (**d**) A4; (**e**) A5; (**f**) A6; (**g**) A7; (**h**) A8; (**i**) A9.

AE characteristic parameters could be used to analyze the fracture behavior of concrete. The AE parameters of nine orthogonal tests were analyzed to evaluate the effects of water–binder ratio, crumb rubber replacement rate and basalt fiber content on the fracture behavior of concrete. The load level is defined as the load at some point divided by the maximum load. The cumulative AE hits and counts vs. load level are shown in Figure 7.

It can be seen from Figure 7 that the fracture process can be divided into three stages according to the curve curvature changes of cumulative AE hits and counts. In Figure 7f, stage II is short and the load level ranges from 0.4 to 0.7, which indicates that rapid crack expansion leads to concrete fracture during the loading process. In Figure 7c,h, the critical point of stage III is at the load level 0.8. The cumulative AE hits and counts increase rapidly in stage III; due to the appearance of macroscopic cracks, the cumulative counts increases sharply when it reaches 2500 and 5000 in Figure 7c,h, respectively, which represents the ultimate fracture of the concrete specimens. In Figure 7a,e,g,i, the critical point of stage III is at the load level 0.9; the cumulative AE hits and counts increase sharply at stage III and the concrete specimen fails. The cumulative AE hits and counts increase slowly in stage II, which indicates that the crack inside the concrete is in a stage of stable expansion. In Figure 7b,d, the critical point of the stage III is at the load level above 0.9. Most AE events occur in stage III, which indicates that most of the damage occurs in this stage.

AE hits could reflect the total and frequency of AE signals; it is constantly used to evaluate AE activity. The AE activity is related to the elastic wave released by the internal damage of concrete. The internal damage of concrete could be characterized by the AE hits. In order to clearly characterize the damage degree of concrete at different load levels, the relative cumulative hits (*RCH*) is defined and calculated by Equation (8). The *RCH* is positively correlated with the internal damage degree of concrete. The greater the *RCH*, the greater the internal damage degree of concrete and the closer the concrete specimen is to failure.

(8) $$RCH=\frac{H_{x}}{H_{total}}$$

where *H_x_* refers to the cumulative hits at the *x* level and *H_total_* refers to the cumulative hits when the specimen is damaged.

According to the curve curvature changes of cumulative AE hits and counts, the fracture process of a concrete specimen is divided into three stages: (I) crack initiation stage, (II) crack expansion stage, and (III) failure stage. The critical point of the crack expansion stage and the fracture stage could be used to evaluate the internal damage degree of concrete before the macroscopic crack appears. The critical points of the fracture stage of nine orthogonal tests were determined based on the visual analysis of AE parameters. The load levels of the critical point of nine groups are about 0.9, so load level 0.9 is selected as the standard. If the critical point is lower than load level 0.9, it indicates that the corresponding concrete specimen has more internal damage and the fracture stage enters the failure stage; if the critical point is higher than load level 0.9, it indicates that the corresponding concrete specimen has less internal damage and the internal crack is still in the expansion stage. In order to clearly reflect the effects of water–binder ratio, crumb rubber replacement rate and basalt fiber content on the fracture process of CRBFC, the relative cumulative hits at load level 0.9 was analyzed by range analysis and variance analysis.

**Figure 8:**
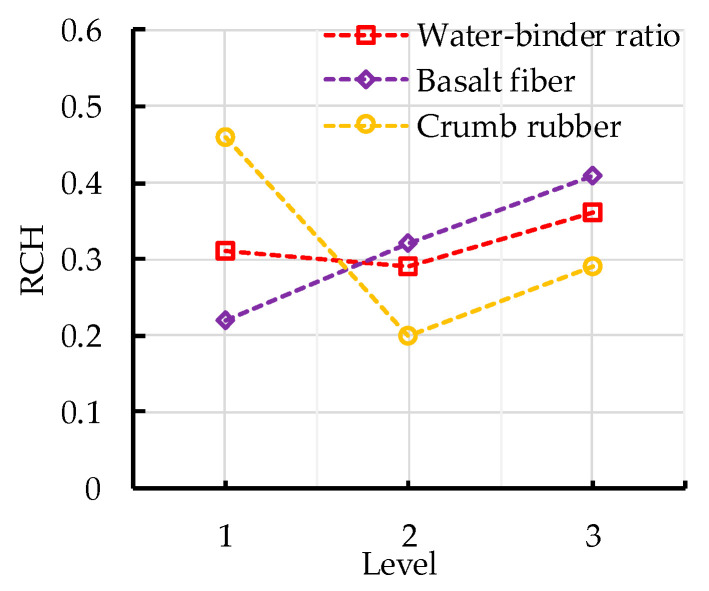
Effects of water–binder ratio, basalt fiber content and crumb rubber replacement rate on *RCH*.

The range analysis of *RCH* is shown in Table 12. It can be seen that the error has least impact on the *RCH*, and the crumb rubber has the greatest impact on the *RCH*, followed by basalt fiber and water–binder ratio. The effects of water–binder ratio, basalt fiber and crumb rubber replacement rate on the *RCH* are shown in Figure 8. The *RCH* increases first and then decreases with the increase in water–binder ratio, which is related to the degree of hydration of cement. The higher the degree of cement hydration, the denser the concrete and the less internal damage. The *RCH* increases with the increase in basalt fiber content. The appropriate amount of basalt fiber can inhibit the initiation and expansion of internal cracks, thus reducing the internal damage degree of concrete. However, excessive basalt fiber will destroy the compacted structure of concrete and cause more damage in the process of pulling out the fiber. The *RCH* decreases first and then increases with the increase in crumb rubber replacement rate. The appropriate amount of crumb rubber can improve the toughness and crack resistance of concrete, and the crumb rubber modified by sodium hydroxide can increase the hydration degree of cement, thus improving the concrete internal compactness. However, excessive crumb rubber will lead to weak bonding strength between the crumb rubber and cement, increasing the risk of internal damage to the concrete. In addition, it can be seen from Table 12 and Figure 8 that the optimal levels of water–binder ratio, basalt fiber content and crumb rubber replacement rate are 0.46, 2kg/m^3^ and 10%, respectively.

The variance analysis of *RCH* is shown in Table 13. It can be seen from the significance level judgment of *RCH* that the *F* values of crumb rubber replacement rate and basalt fiber content are greater than 19.0, which indicates that these two factors have the most significant effect on the *RCH*. The *F* value of crumb rubber replacement rate is greater than basalt fiber content, which means the effect of crumb rubber is more significant. The *F* value of water–binder ratio is between 9.0 and 19.0, which indicates that water–binder ratio has a significant effect on the *RCH*. The results are consistent with those of range analysis.

### 3.2. Fracture Warning and Fracture Mode

#### 3.2.1. Analysis of Fracture Warning

**Figure 9:**
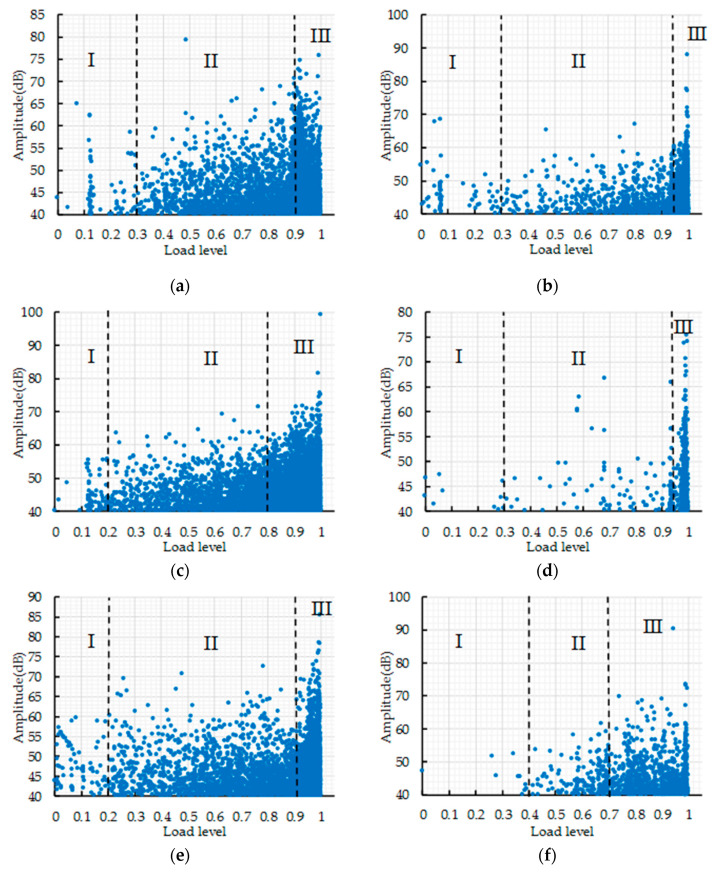

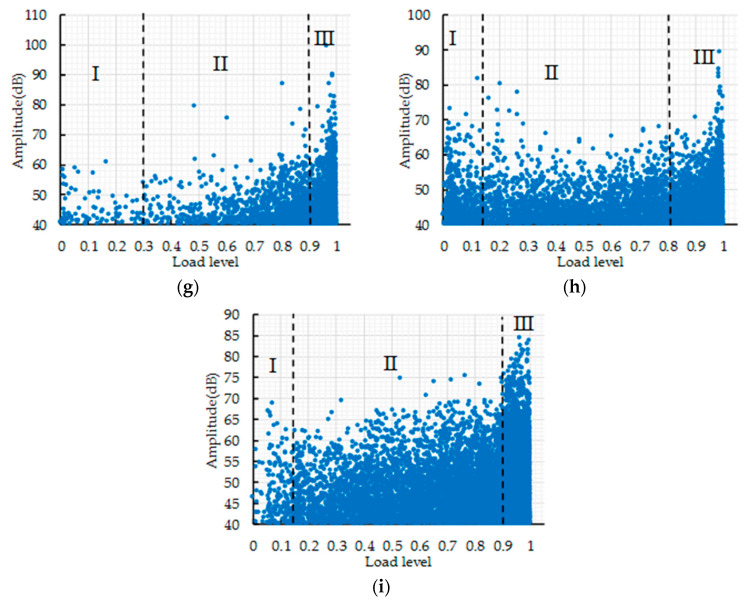
The distribution of AE amplitude values versus load level l: (**a**) A1; (**b**) A2; (**c**) A3; (**d**) A4; (**e**) A5; (**f**) A6; (**g**) A7; (**h**) A8; (**i**) A9.

The AE amplitude value vs. load level is shown in Figure 9. AE amplitude value is divided according to cumulative AE hits and counts. It can be seen from Figure 9 that the AE amplitude has a great correlation with the fracture stage of concrete and the AE signal is less in the first stage, which is related to the less internal damage of concrete under the condition of low load level. In Figure 9f, most of the AE amplitude values are below 50 dB in stage II, the AE amplitude value reaches 60 dB at the boundary of stage II and III, and the AE amplitude value increases sharply for the second time when the macroscopic cracks appear. In Figure 9i, the AE amplitude value increases steadily in stage II and most of the AE amplitude values range from 40 to 60 dB; the AE amplitude value suddenly changes to 75 dB at the critical point of stage III and gradually increases with the increase in load level until the specimen fracture. In Figure 9a, most of the AE amplitude values are between 40 and 60 dB in stage II, the AE amplitude value reaches above 70 dB at the boundary of stage II and III, and, when the specimen is fractured, the AE amplitude reaches above 75 dB. In Figure 9c,e,g,h, most of AE amplitude values range from 40 to 60 dB, the AE amplitude value reaches more than 65 dB at the boundary of stage II and III, and the AE amplitude value reaches 75 dB when macroscopic fracture occurs. It should be noted in Figure 9h that the AE amplitude is more than 65 dB in stage I, which is related to the phenomenon that a large microcrack appears in the concrete and releases a high-strength elastic wave. In Figure 9b, the AE amplitude value appears to mutate at the boundary of stage II and III and increases from below 55 dB to above 60 dB; the AE amplitude value reaches 75 dB when the concrete fractures. In Figure 9d, most of the AE amplitude values are below 55 dB in stage II; the AE amplitude value reaches 65 dB at the boundary of stage II and III, and the AE amplitude increases suddenly for the second time and reaches 75 dB when macroscopic fracture occurs. The number of AE signals increases suddenly when the concrete fracture stage changes and the AE amplitude value increases sharply at the boundary of the fracture stage. Hence, AE amplitude value could be used as an early warning of concrete structure fractures.

#### 3.2.2. Analysis of Fracture Mode

**Figure 10:**
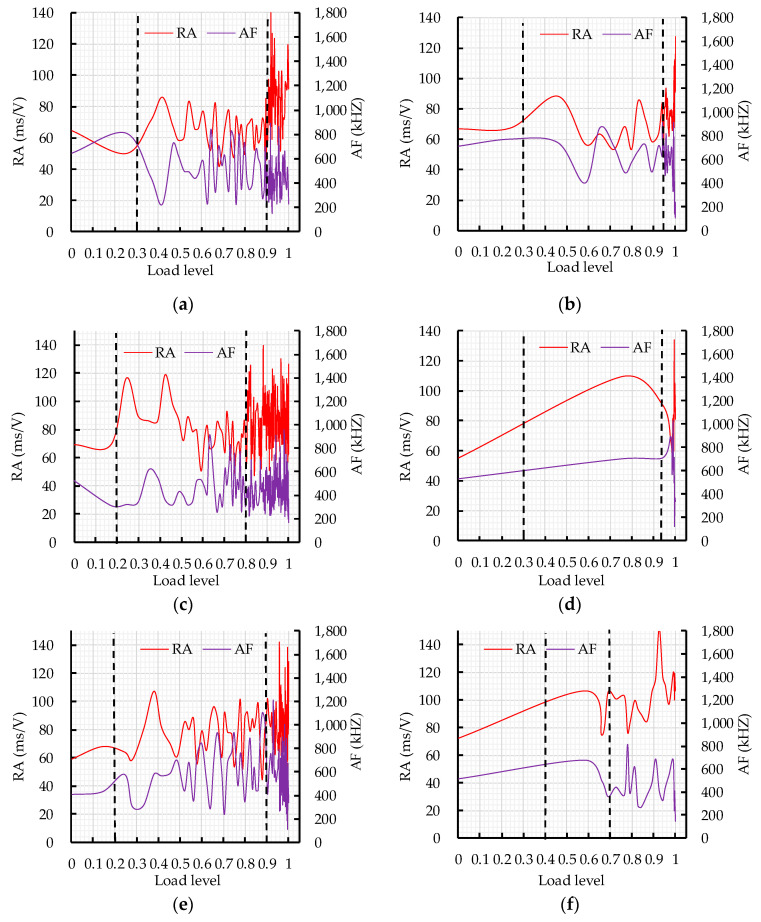

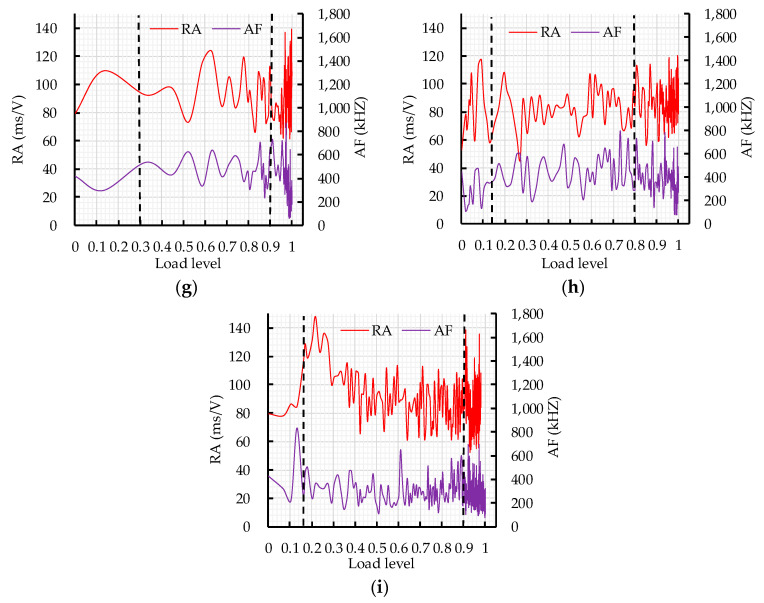
The curve of the RA and AF values versus load level l: (**a**) A1; (**b**) A2; (**c**) A3; (**d**) A4; (**e**) A5; (**f**) A6; (**g**) A7; (**h**) A8; (**i**) A9.

The RA and AF values vs. load level are shown in Figure 10. The fracture mode of CRBFC under flexural conditions was analyzed according to the dynamic change of RA and AF values during the loading process. In order to reduce scattering and make the trend clear, each point on the curve is the moving average of nearly 50 AE hits. In the cracking process of concrete, the logical sequence of crack initiation starts with tensile cracking of the concrete matrix; the fiber friction and pull-out events start to occur as the crack length increases and the shear mode becomes more active [45,46]. Compared with the tensile mode, the shear mode has a higher RA value and lower AF value. As shown in Figure 10, RA and AF values curves fluctuate continuously with the increase in load level. This is related to the dynamic fracture behavior of concrete. In Figure 10a, the RA value increases significantly in the failure stage and the AF value shows a downward trend in the loading process, which indicates that the shear mode dominates the fracture in the failure stage. In Figure 10b, RA and AF values show steady fluctuation in the first two stages and RA value reaches peaks and AF value sharp drops at the moment of fracture. This is related to the transformation of the fracture mode. The sudden change in RA and AF values could reflect the transformation of the fracture mode, which is consistent with the results obtained by Souliout [46]. In Figure 10c, the RA value increases to 110 ms/V and the AF value decreases to 240 kHz in the failure stage compared with the initial RA and AF values, which indicates that the shear mode dominates the fracturing of concrete. In Figure 10d, the RA value shows a rising trend while the AF value changes little in the first two stages; the RA value reaches a peak and AF value sharply drops at the moment of concrete fracture. In Figure 10e, the RA and AF values gradually increase with the increase in load level when the load level is below 0.9, which indicates that a coupling effect of shear mode and tensile mode exists. The shear mode that dominates the concrete fracture is expressed by the increase in the RA value to 110 ms/V and the drop of AF value to below 240 kHz. In Figure 10f, the shear fracture of concrete is expressed by the increase in the RA value to 110 ms/V and the drop of AF value to below 240 kHz. In Figure 10g, the RA and AF values of the initial tensile crack are 80 ms/V and 400 kHz, respectively. The shift from tensile mode to shear mode is expressed by the increase in RA value to 110 ms/V and the drop of AF value to below 240 kHz. In Figure 10h, the RA and AF values of the initial tensile crack are 50 ms/V and 400 kHz, respectively. In the loading process, most of the RA values are above 50 ms/V and most of the AF values fluctuate in the range of 200 kHz and 600 kHz, which is related to the shear effect caused by fiber friction and pulling out. The RA value increases to 110ms/V and the AF value decreases to below 240 kHz at the moment of shear fracture of concrete. In Figure 10i, compared with the initial RA value and AF value, the RA value increases to 110 ms/V and the AF value decreases to below 200 kHz in the failure stage, which represents the transformation from tensile mode to shear mode. In the loading process, the initial crack in CRBFC is a tensile crack and the RA and AF values of the initial crack are below 80 ms/V and above 400 kHz, respectively. The shear mode that dominates the failure stage of CRBFC is expressed by the increase in RA value to 110 ms/V and the drop in AF value to 240 kHz.

## 4. Conclusions

In this paper, the effects of water–binder ratio, basalt fiber content and crumb rubber replacement rate on the mechanical properties and fracture behavior of CRBFC were studied by orthogonal testing. The fracture process of CRBFC under a flexural load was monitored by AE technology. Based on the experimental results, the following conclusions can be drawn:

1. Adding 0.25 M sodium-hydroxide-solution-modified crumb rubber can effectively improve the bonding strength between crumb rubber and cement, thus enhancing the compressive strength and flexural strength of CRBFC.

2. Based on the orthogonal analysis results of the compressive strength, flexural strength and *RCH*, the optimal levels of water–binder ratio, basalt fiber content and crumb rubber replacement rate are 0.46, 2kg/m^3^ and 10%, respectively.

3. The positive synergistic effects of basalt fiber and crumb rubber can effectively improve the mechanical properties and fracture behavior of concrete and increase the practicability of basalt fiber and crumb rubber in the field of concrete.

4. AE characteristic parameters have a great correlation with the fracture stages of concrete, and the fracture stages of CRBFC can be accurately divided according to the cumulative AE hits and counts. The mutation of AE amplitude value symbolizes the transformation of the fracture stage of concrete. AE amplitude can be used as an early warning of concrete structure fracture.

5. The fracture mode of CRBFC is dominated by the shear mode at the failure stage. In addition, RA and AF values can be used as the evaluation index of concrete fracture mode, which could assist in the assessment of concrete damage.

## Figures and Tables

**Table 1:** The physical and mechanical properties of cement.

Density (kg/m^3^)	Specific Surface Area (m^2^/kg)	Setting Time (min)		Compressive Strength (MPa)	Flexural Strength (MPa)
		Initial Setting	Final Setting		
3160	385	91	145	62.2	9.1

**Table 2:** The chemical composition of cement.

Material	Chemical Composition (%)					
	SiO_2_	Al_2_O_3_	Fe_2_O_3_	CaO	MgO	SO_3_
Cement	22.60	5.60	4.30	62.70	1.70	2.50

**Table 3:** The performance indicator of basalt fibers.

Type	Length (mm)	Diameter (μm)	Linear Density (tex)	Tensile Strength (MPa)	Elastic Modulus (GPa)	Breaking Strength (N/tex)	Elongation (%)
Basalt fiber	22	23	2392	2836	62	0.69	3

**Table 4:** Parameters in the orthogonal experimental design.

Levels	Factors		
	Water–Binder Ratio	Basalt Fiber (kg/m^3^)	Crumb Rubber (%)
1	0.43	2	5
2	0.46	4	10
3	0.49	6	15

**Table 5:** Specific orthogonal experimental table.

Test Number	Parameters				Result Value
	Water–Binder Ratio	Basalt Fiber (kg/m^3^)	Crumb Rubber (%)	Error	
1	0.43(1)	2(1)	5(1)	1	y_1_
2	0.43(1)	4(2)	10(2)	2	y_2_
3	0.43(1)	6(3)	15(3)	3	y_3_
4	0.46(2)	2(1)	10(2)	3	y_4_
5	0.46(2)	4(2)	15(3)	1	y_5_
6	0.46(2)	6(3)	5(1)	2	y_6_
7	0.49(3)	2(1)	15(3)	2	y_7_
8	0.49(3)	4(2)	5(1)	3	y_8_
9	0.49(3)	6(3)	10(2)	1	y_9_

**Table 6:** The mix proportion of CRBFC.

Mix ID	Water-Binder Ratio	Water (kg/m^3^)	Cement (kg/m^3^)	Fine Aggregate (kg/m^3^)	Coarse Aggregate (kg/m^3^)	Basalt Fiber (kg/m^3^)	Crumb Rubber (kg/m^3^)	Modifier
A1	0.43	180	418.60	563.78	1204.88	2	12.46	NaOH
A2	0.43	180	418.60	534.11	1204.88	4	24.92	NaOH
A3	0.43	180	418.60	504.43	1204.88	6	37.37	NaOH
A4	0.46	180	391.30	541.24	1220.97	2	25.25	NaOH
A5	0.46	180	391.30	511.17	1220.97	4	37.37	NaOH
A6	0.46	180	391.30	571.31	1220.97	6	12.62	NaOH
A7	0.49	180	367.35	517.08	1235.10	2	39.31	NaOH
A8	0.49	180	367.35	577.91	1235.10	4	12.77	NaOH
A9	0.49	180	367.35	547.50	1235.10	6	25.54	NaOH
B1	0.43	180	418.60	504.43	1204.88	6	37.37	–
C1	0.43	180	418.60	593.45	1204.88	–	–	–
C2	0.46	180	391.30	601.38	1220.97	–	–	–
C3	0.49	180	367.35	608.33	1235.10	–	–	–

**Table 7:** The experimental results of compressive strength and flexural strength tests.

Test	Test Number	Parameters				Compressive Strength (MPa)	Flexural Strength (MPa)
		Water-Binder Ratio	Basalt Fiber (kg/m^3^)	Crumb Rubber (%)	Error		
Test Group	A1	0.43	2	5	1	40.6	3.82
	A2	0.43	4	10	2	39.8	3.79
	A3	0.43	6	15	3	33.7	3.37
	A4	0.46	2	10	3	43.4	4.16
	A5	0.46	4	15	1	37.4	3.87
	A6	0.46	6	5	2	38.9	3.25
	A7	0.49	2	15	2	39.5	3.98
	A8	0.49	4	5	3	38.1	3.40
	A9	0.49	6	10	1	38.3	3.56
Control Group	B1	0.43	6	15	–	31.8	2.78
	C1	0.43	–	–	–	29.6	2.94
	C2	0.46	–	–	–	31.5	3.02
	C3	0.49	–	–	–	31.1	2.81

**Table 8:** Range analysis for compressive strength of CRBFC.

Factor	Level			Range
	1	2	3	
Water–binder ratio	k_1_ = 38.03	k_2_ = 39.90	k_3_ = 38.63	1.87
Basalt fiber	k_1_ = 41.17	k_2_ = 38.43	k_3_ = 36.97	4.20
Crumb rubber	k_1_ = 39.20	k_2_ = 40.50	k_3_ = 36.87	3.63
Error	k_1_ = 38.77	k_2_ = 39.40	k_3_ = 38.40	1.00

**Table 9:** Variance analysis for the compressive strength of CRBFC.

Factor	*SS*	*df*	*MS*	*F* Value	*F* Critical Value	Significance
Water–binder ratio	5.449	2	2.724	3.55	F_0.05_(2,2) = 19.0	less significant
Basalt fiber	27.262	2	13.631	17.75	F_0.1_(2,2) = 9.0	significant
Crumb rubber	20.336	2	10.168	14.24	F_0.25_(2,2) = 3.0	significant
Error	1.536	2	0.768	–	–	–

**Table 10:** Range analysis for flexural strength of CRBFC.

Factor	Level			Range
	1	2	3	
Water-binder ratio	k_1_ = 3.66	k_2_ = 3.76	k_3_ = 3.65	0.11
Basalt fiber	k_1_ = 3.99	k_2_ = 3.69	k_3_ = 3.39	0.60
Crumb rubber	k_1_ = 3.49	k_2_ = 3.83	k_3_ = 3.74	0.35
Error	k_1_ = 3.75	k_2_ = 3.67	k_3_ = 3.64	0.10

**Table 11:** Variance analysis for the flexural strength of CRBFC.

Factor	*SS*	*df*	*MS*	*F* Value	*F* Critical Value	Significance
Water-binder ratio	0.022	2	0.011	1.32	F_0.05_(2,2) = 19.0	least significant
Basalt fiber	0.532	2	0.266	32.06	F_0.1_(2,2) = 9.0	most significant
Crumb rubber	0.192	2	0.096	11.56	F_0.25_(2,2) = 3.0	significant
Error	0.017	2	0.008	–	–	–

**Table 12:** Range analysis for *RCH*.

Factor	Level			Range
	1	2	3	
Water-binder ratio	k_1_ = 0.31	k_2_ = 0.29	k_3_ = 0.36	0.07
Basalt fiber	k_1_ = 0.22	k_2_ = 0.32	k_3_ = 0.41	0.19
Crumb rubber	k_1_ = 0.46	k_2_ = 0.20	k_3_ = 0.29	0.26
Error	k_1_ = 0.31	k_2_ = 0.33	k_3_ = 0.33	0.02

**Table 13:** Variance analysis for *RCH*.

Factor	*SS*	*df*	*MS*	*F* Value	*F* Critical Value	Significance
Water-binder ratio	0.007298	2	0.00365	10.26	F_0.05_(2,2) = 19.0	significant
Basalt fiber	0.056094	2	0.02805	78.97	F_0.1_(2,2) = 9.0	most significant
Crumb rubber	0.103780	2	0.05189	145.99	F_0.25_(2,2) = 3.0	most significant
Error	0.000711	2	0.00036	–	–	–
